# A Saturday Night at the London Hospital

**Published:** 1889-06-22

**Authors:** 


### The Hospital Workshop.

### No. III.—A SATURDAY NIGHT AT THE LONDON HOSPITAL.

(BY OUR SPECIAL COMMISSIONER.)

In his book "How the Poor Live," Mr. Sims has told us a good deal about life in the East End of the city that is the greatest and richest in the world. There could be no better way of gaining an insight into some of its sterner realities than by a Saturday night visit to the great hospital of that quarter, the receiving-room of which is the centre of a stream of sick and injured people, for the most part bearing an unmistakable stamp of poverty and debauchery. Drink is their curse: bad public-house liquor, drugged beer, new and raw spirits. On Saturday wages are paid, and then follows a drinking bout. Friends fight; strangers fight; men, women, and children are variously damaged in the huge rough-and-tumble scrambling dissipation; and the victims of the alcoholic poison pour into the "London" until two or three next morning. After a few hours of this sort of thing one feels inclined to join Sir Wilfrid Lawson off-hand in his crusade against the publican, to whom much of this crime, degradation, disease, and suffering may be traced.

All patients applying for treatment, medical or surgical, are directed to the receiving-room, which lies close to the main entrance. The lesser cases are seen to at once, and urgent ones sent on to the wards. There is a special officer, who attends from 10 a.m. to 6 p.m. every day except Sunday. Two of the residents on "full duty"—a house surgeon and a house physician—follow from 6 to 9 p.m., and again from 11 to 1 p.m.; on Saturdays, 11 to 2 p.m. They are relieved by "half-duty" men from 9 to 11 p.m.; and the night shifts (from 1 to 9 a.m.) are shared by the others of the resident staff in turn. Under supervision, much of the actual work is done by "dressers" and "clerks," chosen from among the students. There is a sister in charge and a nurse for each of the consulting-rooms, male and female.

When your commissioner called about seven o'clock last Saturday evening, the house surgeon on full duty, after exchanging a few words, led the way to the men's side-room, which was filled by the following patients:

Case 1.—A boy of seven, finger-nail crushed off.

Case 2.—Young man; lean visage; bad cough. (To see house physician.)

Case 3.—Burly waterman, complaining of throat. Has a black eye and is far from sober.

Case 4.—Pale, elderly man, who has "started a vein in his leg." This is a common accident with varicose veins. The flow of blood has been arrested by the pressure of a broad plaster. Rest means everything in the cure of these cases, but patient says he cannot leave off work.

Case 5.—A sober mechanic. Had a bad finger (whitlow) which he attributes to working in acid some weeks ago.

Case 6.—Middle-aged man under the influence of drink. Complains of severe bleeding from the nose. Is told to press his forehead with a large lump of ice which he repeatedly lets fall into the basin, much to the amusement of the other patients. Eventually both nostrils have to be plugged with lint.

Case 7.—A foreign Jew with a swollen lip. It was difficult to say whether his curious exaggeration of manner is due to alcohol or not. He says, "One of your doctors do dis vid the instrument. He took me out de tooth dis morning. He is not fit to be here. He is von butcher." The lip is slightly grazed, a thing which will happen now and then in drawing teeth. The patient has been twenty years in England, and does not remember the name of his birth-place, but knows it is somewhere in Hungary. Foreign Jews abound in Whitechapel, and great numbers of them come to this hospital, where special wards are set apart for their use.

Case 8.—A white-faced shoemaker, elderly and bald, who has had just enough drink to make him troublesome. He shows a skin eruption, of obscure origin, on the arm, and is handed over to the house physician.

Case 9.—Young man of two or three and twenty, who looks like a prize-fighter. His hair is so closely cropped as to resemble down, and on the scalp are a number of white scars. One of them, two or three inches long, and exactly in the middle line, runs into the forehead. It looks as if he had begun to part his hair in the middle and had suddenly altered his mind. His nose is broken, jaw heavy, neck like a prize bull, and frame muscular. He comes to-night with a suspicious-looking injury to the back of the right hand, just under the third finger, a place that is often damaged in striking with the clenched fist. At present the swelling prevents one making out for certain whether the bone is broken or not, so a splint is fitted on and he is told to come again next week.

Case 10.—A splendidly muscular fellow, who was treated here a few days ago for a sprained shoulder (has a fresh cut on the eyebrow).

Case 11.—Engineer with crushed finger.

Case 12.—Scald; boy playing with kettle.

Case 13.—Foreign body in eye. Irish labourer.

Case 14.—Painful leg. (Medical case.)

Case 15.—Another shoemaker, an undersized young man, the picture of unhealth. While at work some hours before he drove an awl deep into his palm. (He has staunched the bleeding with tobacco, an efficient if not very cleanly plan.)

This clears the male side for the time being, and then we cross over to the women's room.

Case 16.—A little girl about three. The mother says a cart fell on the child that morning. Further questioned, says the cart was "a very heavy one. Indeed, you might almost call it a van." The child is able to walk across the room, despite her mother's exaggeration and distress, which seem to be largely prompted by whisky.

Case 17.—A Scotch girl who says that five months ago, when in service at Newcastle, she was bitten in several places by a curly black retriever dog. She went to a hospital, but did not have the bites cauterised (wisely, for the proceeding is utterly useless, as a rule). The scar on her face has since been painful. On the cheek is the mark of an old wound, red and raised, and apparently very tender. The patient, however, is highly nervous, and shrinks away before the place is touched. It is pretty safe, therefore, to assume that her (unexpressed) fears are groundless, and a nerve sedative is prescribed, with directions to come back should she not get better. By the way, her christian-name is Flaminia.

Case 18.—Girl with cut arm—a couple of stitches.

Case 19.—Woman with sprained ankle (alcoholic). The foot is done up with strips of plaster and bandage.

Case 20.—A woman who complains of pain in the front of her foot, and insists strongly on the fact that it never occurred before. She has a flat foot, and is suffering from an affection that the German writers have dignified by the name of "anterior metatarsalgia." This fact, however, is not imparted to the patient, as it would be giving her an unfair advantage over her neighbours to send her home in possession of a disease with so imposing a name.

The great number of cases are surgical, and the men far outnumber the women. There are still four or five of the latter to be seen, but about this point your commissioner gave up counting. The room being now empty, the house surgeon is going off to attend to a case sent into the wards an hour previously. Let us go with him.

Down long corridors with sudden twists and turns, and then through rows and rows of beds until we reach one where a lad of fifteen is lying with his head swathed in bandages. He has been stabbed in several places by another boy about his own age, and is able to give a clear account of the affair. "Is it hurting you much?" "Yiss, sir," he answers in a shrill voice. "Do you know the boy that did it?" "Yiss, Bishop." "What did he do it for?" "Why, it was this way. He hit me in the face with a turnip, Wednesday night, and I niver seed 'im again afore to-night. I says, 'You ought to get a smack in the nose for what you done. You don't know but what you might have blinded me.' He up and says, 'Well! I'll do something better nor take your eye,' and with that he stabbed me in the side and ear."

It is now after eight o'clock; the gas is turned down and the patients have gone to rest. But this does not prevent one or two curious heads from peering round the blue-striped curtains to see what is going on. The dressings and lotions are placed on a table at the foot of the bed, round which a screen is drawn. One sister holds the lamp, another takes charge of the patient, while a third looks after the instruments and sponges. Then comes the examination. The upper third of the ear is cut through and left hanging, and a nasty wound, three or four inches in length, runs backwards along the scalp. The house surgeon, wishing to avoid chloroform, uses cocaine solution to deaden the pain. He then fixes the severed ear carefully together with a number of catgut stitches, after which the wound is dusted with iodoform (a powerful antiseptic), and the whole padded and wrapped up in wood wool, which is soft, warm, and absorbent. Beyond crying out now and then the boy has borne these somewhat painful attentions very bravely, and when his head has been securely fastened up, remarks, "My side is the wustest." That is not the fact, however, luckily for himself; for it turns out to be a simple cut, half an inch long, which does not reach the underlying rib.

As this will be a "police" case, requiring evidence, the house surgeon enquires for the patient's clothes, in order to compare the marks on them with the wound. The boy's keen interest shows no signs of flagging, and he reminds the sister that his coat was "bottle-green."

Then our busy resident was called off to a distant ward to tap a chest so distended with fluid as to dangerously hamper the breathing. The patient is leaning forwards in bed, weak, tremulous, and exhausted; his voice reduced to a mere whisper. No time is lost. By means of a hand-pump, an assistant exhausts the air from a large Winchester bottle, and into this the matter flows through a tube, one end of which fits on a hollow needle that is plunged between the ribs. On leaving the ward after the operation, the house surgeon was stopped by a big bearded man who had been waiting quietly outside. He introduced himself as an inspector of police, and said he had come to enquire about a boy who had been stabbed that evening. He wanted to know whether the wounds were dangerous, and when the boy would be able to appear in court, and, producing an ordinary pocket-knife with a blade about four inches long, "Do you think the injuries could have been inflicted with this weapon?" It seems the culprit had been taken into custody, but as at the time of writing the case has not been before the magistrates, it will be well to refrain from any comment. Your commissioner has a wholesome dread of wandering into the mysterious maze of contempt of court.

The use of the knife is becoming a far too common offence. For instance, one of the sisters remarked that three cases of stabbing by boys who had been playing at "Jack the Ripper" were brought to her ward in a week. These facts show how that terrible example is likely to work upon a certain order of minds.

It is now time to pay another visit to the receiving-room. There has been a decided lull, for between nine and ten o'clock not more than six cases have been entered, and sixteen only followed during the next hour. Later on, however, when the public-houses close, the rush will commence, and the ball be kept rolling until two or three in the morning. Curiously enough, the first man we see has been stabbed in the back of the thigh. He says he had a "bit of a bother" with an entire stranger, who knocked him down and decamped. Some time afterwards our patient discovered he had been stabbed. This tale is most likely untrue. These people often tell a cock and bull story, prompted either by a misguided instinct of loyalty to their "pals," or else by the fear of further vengeance.

Next, is a little boy with an eyebrow cut open by a fall on the kerbstone. He is brought by a pale and agitated youth of seventeen, who explains that he "ain't no relative, but only a friend as lives in the same 'ouse," in more direct language a lodger.

Here is a man with a broken kneecap. He seems to be in a curious half-dazed kind of drunken state, and after examination is sent on to the wards. The leg is first fastened securely to a kind of light pillow, which protects the limb, and acts as a temporary splint.

His neighbour has a black eye, is splashed with mud, and is very drunk. At the back of his head he shows a lacerated wound, which requires a few stitches. His story runs somewhat as follows: "Me and a friend was out together. We was out, and we was sociable together. He is a friend of mine—a very great friend—and we was out together. We had a drop of drink. We got to words together, and he hits me over. He's a very great friend of mine, and I won't say nothing more about it, so long as he don't do it to no one else." (Sudden change of tone.) "But I'll make him pay for this! I'll lock him up for this job! He ain't a goin' to knock me about like this for nothink! Not me! Ain't good enough!" At length he is persuaded to go away, still ringing the changes of alternate vengeance and forgiveness on the friend of his bosom.

Here is a stalwart carman, limping in slowly. In jumping backwards out of his van he fell with his right leg doubled up under him. The limb is so swollen as to make the nature of the injury doubtful, but no bones appear to be broken.

One man, very drunk, has a broken arm put up in splints. Says "this is a funny place, a very funny place. Nobody won't listen to me." Wants to embrace the dresser who has set his arm, and offers repeatedly to stand drinks all round. Refuses point-blank to take his letter for further attendance as an out-patient. Says he doesn't want to come again. Why should he? And so he rambles along, until at last the letter is stuffed into his pocket and the porter leads him complainingly away.

Amid all this saddening work it is pleasant to note one redeeming feature, and that is how little the patients swear. It is just as natural, be it remembered, for many of them to use strong language as it is for most people to refrain, and the fact shows how a sense of propriety asserts itself even in the depths of that awful quagmire of drunkenness.

During a single hour on a weekday eighty applicants were counted; but that, of course, was an exceptionally busy time. Three hundred and forty were reported another day, and over 21,000 in the three months.

So that one might go on describing cases until further orders, but enough has been given to show the nature of the work. Let the reader fill up the picture by imagining a crowd of people pouring in until the small hours of Sunday morning with cuts, bruises, burns, dislocations, broken bones, and other sudden ills that flesh is heir to. The strain upon the nurses, dressers, and resident officers will also be partly realised. A last word may be added about a case on the female side. It is that of a grisled woman, some fifty years of age, dressed in rusty black, and talking at a great rate. She says: "When I went to get the kettle mended in Brick-lane, you see I fell down and hurt my knee. I says to my husband 'I'm all right,' I says, 'I am. Don't take me to no 'orspital.' And then I goes home; but my leg it gets worse, and my 'usband brings me here. I've been in service all my life, and I never had nothing like this before." There is not much the matter with the knee, and the house surgeon orders a bandage. However, the patient does not chime in with that suggestion, but jumping up forthwith, she addresses the nurse in this wise: "No, my dear, no bandage for me, thank you. I shall be all right, I shall. I won't have no bandage, thank you, my dear. Good-night." And then she stalked off with firm stride and heavy step, doubtless to rejoin her boon companions.

